# Targeting TGFβ signal transduction for cancer therapy

**DOI:** 10.1038/s41392-020-00436-9

**Published:** 2021-01-08

**Authors:** Sijia Liu, Jiang Ren, Peter ten Dijke

**Affiliations:** Oncode Institute and Department of Cell and Chemical Biology, Leiden University Medical Center, Einthovenweg 20, 2300 RC Leiden, The Netherlands

**Keywords:** Cell biology, Molecular medicine

## Abstract

Transforming growth factor-β (TGFβ) family members are structurally and functionally related cytokines that have diverse effects on the regulation of cell fate during embryonic development and in the maintenance of adult tissue homeostasis. Dysregulation of TGFβ family signaling can lead to a plethora of developmental disorders and diseases, including cancer, immune dysfunction, and fibrosis. In this review, we focus on TGFβ, a well-characterized family member that has a dichotomous role in cancer progression, acting in early stages as a tumor suppressor and in late stages as a tumor promoter. The functions of TGFβ are not limited to the regulation of proliferation, differentiation, apoptosis, epithelial–mesenchymal transition, and metastasis of cancer cells. Recent reports have related TGFβ to effects on cells that are present in the tumor microenvironment through the stimulation of extracellular matrix deposition, promotion of angiogenesis, and suppression of the anti-tumor immune reaction. The pro-oncogenic roles of TGFβ have attracted considerable attention because their intervention provides a therapeutic approach for cancer patients. However, the critical function of TGFβ in maintaining tissue homeostasis makes targeting TGFβ a challenge. Here, we review the pleiotropic functions of TGFβ in cancer initiation and progression, summarize the recent clinical advancements regarding TGFβ signaling interventions for cancer treatment, and discuss the remaining challenges and opportunities related to targeting this pathway. We provide a perspective on synergistic therapies that combine anti-TGFβ therapy with cytotoxic chemotherapy, targeted therapy, radiotherapy, or immunotherapy.

## Introduction

Transforming growth factor-β (TGFβ) belongs to a family of multi-functional cytokines that includes TGFβs, bone morphogenetic proteins, activins, nodal, growth and differentiation factors, inhibins, lefty, and anti-Mullerian hormone. Members of this family are key regulators of embryonic development, tissue homeostasis, and regeneration, and their malfunction has been implicated in cancer, fibrosis, immune diseases, and many other pathologies.^1–4^ In this review, we focus on TGFβ, which is the prototypical member of the family. There are three highly structurally related mammalian TGFβ isoforms, that is, TGFβ1, TGFβ2, and TGFβ3.^5,6^ In vitro studies showed that all three isoforms elicit similar biological effects, with differences in potency in certain cell types.^7^ Mouse studies in which each specific isoform was knocked out revealed strikingly different phenotypes, indicating nonredundant in vivo functions for these three TGFβ isoforms. TGFβ1-deficient mice either die of vascular defects during embryogenesis or autoimmune disease postnatally.^8,9^ Mice deficient in TGFβ2 have defects in cardiac septation and valve remodeling,^10^ and mice lacking TGFβ3 have pulmonary defects and cleft palate.^11,12^ TGFβ1 is the most abundant and studied isoform, and it is particularly highly enriched in platelets and bone.^13,14^

**Fig. 1:**
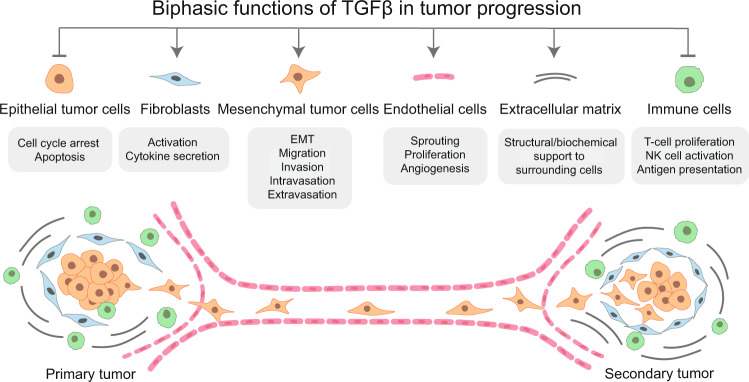
Biphasic functions of TGFβ during tumor progression. TGFβ acts as a tumor suppressor in the initial stage of tumor progression by inducing cell cycle arrest and apoptosis of normal and pre-malignant epithelial cells. Upon activation of oncogenes and/or inactivation of tumor suppressor genes, tumor cells become insensitive to the TGFβ-induced cytostatic effects and undergo uncontrolled proliferation. TGFβ produced by tumor cells, fibroblasts, immune, and endothelial cells in the tumor microenvironment (TME) can trigger cancer cells to undergo an epithelial-to-mesenchymal transition (EMT). Thereby, late-stage cancer cells acquire the ability to escape from the primary niche, intravasate into the circulation, extravasate and localize to distant sites, and progress to form secondary tumors. Reciprocal TGFβ signaling between cancer cells and the TME contributes to cancer progression by activating cancer-associated fibroblasts (CAFs), stimulating angiogenesis, promoting protumor cytokine secretion, increasing extracellular matrix deposition, and evading an immune attack. In the metastatic sites, the mesenchymal tumor cells can undergo a mesenchymal-to-epithelial transition. Thus, thereby change back into an epithelial phenotype, which enables rapid outgrowth

TGFβ signaling has attracted the interest of cancer biologists because of its numerous roles in regulating cancer cell functions, including cell cycle progression, apoptosis, adhesion, and differentiation.^3,15^ The biphasic functions of TGFβ during cancer progression on tumor cells and other cells in the tumor microenvironment (TME) are summarized in Fig. 1. In different cell types and/or conditions, TGFβ can have different, even opposite, effects.^16^ In normal and premalignant cells, TGFβ predominantly acts as a tumor suppressor by inhibiting cell proliferation, promoting apoptosis, and maintaining genome stability.^17^ However, tumor cells can adapt to or selectively bypass the suppressive functions of TGFβ; they utilize TGFβ’s promotional roles to obtain a growth advantage and undergo processes, such as the epithelial-to-mesenchymal transition (EMT), that enable their migration, invasion, intravasation, and extravasation.^18,19^ Moreover, TGFβ can create a beneficial TME by acting in a paracrine manner to activate cancer-associated fibroblasts (CAFs), promote angiogenesis, produce extracellular matrix (ECM), and suppress anti-tumor immune reaction to trigger cancer progression and promote metastasis.^3,4,20^

Many preclinical results from in vitro cell models and in vivo animal models have shown the great potential of anti-tumor therapeutics with TGFβ-neutralizing antibodies and ligand traps that block the interaction of TGFβ with its receptors or selective small-molecule TGFβ receptor kinase inhibitors.^21,22^ However, rendering these pharmacological agents suitable for approved clinical use continues to be a challenge. Because TGFβ shows highly pleiotropic actions, in addition to the tumor itself, healthy tissues can be affected by the systemic inhibition of TGFβ, which can lead to unwanted side effects and safety concerns.^23^ Therefore, we need to better understand the underlying molecular mechanisms by which TGFβ signaling controls normal and malignant processes. Moreover, insight into stratifying tumor patients using biomarkers for selecting patients that may benefit from TGFβ targeting is urgently needed.^24,25^ In this review, we discuss recent clinical advancements and bottlenecks in anti-TGFβ cancer treatment, and provide perspectives of combined treatment to overcome chemo/targeted/radio-therapy resistance and increase the efficiency of immunotherapy responses in cancer patients.

## Bioavailability and activation of TGFβ ligands

**Fig. 2:**
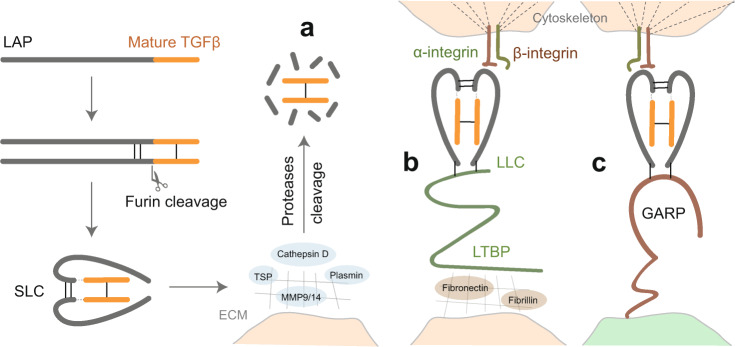
A schematic representation of the activation of latent TGFβ. The pro-TGFβ precursor is synthesized in the rough endoplasmic reticulum. It consists of an N-terminal signal peptide, latency-associated peptide (LAP), and a mature C-terminal TGFβ fragment. After cleavage by the convertase furin in the Golgi complex, the LAP dimer binds to mature TGFβ noncovalently, preventing its binding to cell surface receptors, and forms the small latent complex (SLC). There are three major mechanisms for activation of latent TGFβ. **a** Proteases (e.g., cathepsin, plasmin, matrix metalloproteinase 9/14 (MMP9/14)) in the extracellular matrix (ECM) cleave LAP and release active TGFβ. Also, thrombospondin (TSP) can induce activation by direct binding to LAP. **b** SLC is anchored to the ECM proteins (e.g., fibronectin and fibrillin) via latent TGFβ-binding protein (LTBP) and forms the so-called large latent complex (LLC). Active TGFβ can be released by cell contraction upon the interaction between LAP and integrins. **c** SLC binds to glycoprotein A repetition predominant protein (GARP) on the cell surface and can also mediate the release of active TGFβ upon interaction with integrins

TGFβ is synthesized as a large precursor protein in the rough endoplasmic reticulum, it consists of a signal peptide, a large N-terminal pro-segment termed latency-associated peptide (LAP), and a short C-terminal mature peptide.^26,27^ The pro-peptide assembles into a homodimer in which the LAP portions link via two disulfide bonds and the mature TGFβ moieties’ interaction is stabilized through a disulfide bond.^28^ After cleavage of the precursor protein by the convertase furin in the Golgi complex, the LAP portions encircle the mature portions to form the small latent complex (SLC) and shield the mature TGFβ from binding to its receptors (Fig. 2).^29,30^

For the activation of latent TGFβ, the mature TGFβ portions need to dissociate from the LAP portions. TGFβ can be activated in vitro upon heating or treating with a mild acid (pH 4.5) to dissociate the LAP portion from the mature protein.^31,32^ In addition, reactive oxygen species can activate TGFβ in vitro by destroying the ability of LAP to bind to mature TGFβ via oxidative modification.^33–35^ Several ECM serine proteases (plasmin/plasma kallikrein/cathepsin D) and matrix metalloprotease 9/14 (MMP9/14) can release active TGFβ via the proteolytic cleavage of LAP in vivo (Fig. 2a).^3,31,36^ Thrombospondin (TSP) is another activator of latent TGFβ that is present in the ECM; upon direct binding to LAP, TSP can induce the release of active TGFβ.^37^ In addition, the latent TGFβ binding protein (LTBP) can covalently bind to LAP, which facilitates the deposition of the SLC in the ECM and forms the large latent complex (LLC).^38,39^ LLC can form a covalent interaction with specific ECM proteins such as fibrillin and fibronectin via the N-terminal domain of LTBP (Fig. 2b).^39,40^ Moreover, the latent TGFβ can also interact with the transmembrane glycoprotein A repetition predominant protein (GARP), which is expressed on the cell surface of regulatory T (T_reg_) cells, platelets, and endothelial cells to facilitate latent TGFβ activation (Fig. 2c).^41^ Finally, integrins, which are cell adhesion receptors that control cell proliferation, survival migration, and invasion, also have a pivotal role in the activation of latent TGFβ.^42,43^ Integrins comprise α and β heterodimeric subunits that are both type I transmembrane receptors and are expressed in a wide range of cells.^42^ Certain integrins have been demonstrated to interact with the Arg-Gly-Asp (RGD) motif of LAP and lead to the mechanical release of LAP by cellular contractions.^44–46^

## Functions of TGFβ signaling pathways during tumor progression

**Fig. 3:**
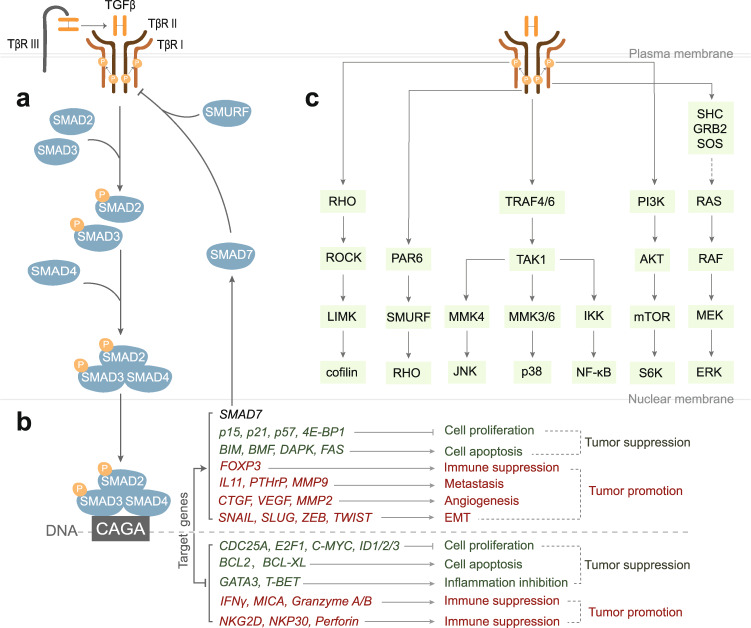
Schematic of the TGFβ-induced canonical SMAD and noncanonical signaling pathways. **a** TβRIII presents TGFβ to TβRII. Thereafter, ligand occupied TβRII recruits and phosphorylates TβRI to trigger intracellular TGFβ signaling pathways. In the canonical pathway, activated TβRI phosphorylates SMAD2/3 and stimulates the formation of heteromeric complexes with SMAD4. These complexes are translocated into the nucleus and regulate target gene expression. One of the TGFβ/SMAD-induced target genes is *SMAD7*, of which the gene product participates in a negative feedback loop to regulate the duration and intensity of TGFβ signaling by recruiting E3 ubiquitin ligase SMURF to TβRI. **b** TGFβ pathway target genes relevant for tumor suppression are listed in green, and the target genes that encode proteins involved in the tumor promotion are listed in red. **c** TGFβ can also activate many noncanonical pathways, including RHO, JNK, p38, NF-κB, AKT, and ERK signaling components

Regarding the canonical TGFβ pathway, active TGFβ initially binds to the low-affinity accessory TGFβ type III receptor (TβRIII), also named betaglycan, which is abundant on the surface of many cell types (Fig. 3a). Betaglycan can present TGFβ to the TGFβ receptor complex, which is present at low levels but high affinity, thereby facilitating signaling.^47^ The TGFβ2 isoform is, in particular, dependent on TβRIII for signaling. Consistent with this notion, endothelial cells that do not express TβRIII show weak responsiveness to TGFβ2 compared with that of TGFβ1 and TGFβ3.^7^ The TGFβ receptors complex is a tetramer consisting of two paired transmembrane serine/threonine protein kinases: two TβRIs and two TβRIIs.^48–50^ TGFβ binding triggers TβRII to transphosphorylate TβRI at specific serine/threonine residues that are located in the intracellular juxtamembrane region enriched with glycine and serine residues (the GS domain).^51,52^ After the extracellular signal is successfully transduced across the plasma membrane, activated TβRI initiates intracellular signaling by phosphorylating SMAD2/3 at their two extreme C-terminal serine residues.^53,54^ Thereafter, phosphorylated SMAD2/3 dissociates promptly from TβRI and assemble into a heteromeric complex with SMAD4. Subsequently, the SMAD2/3–SMAD4 complex can translocate into the nucleus to activate or repress the expression of target genes.^55,56^ *SMAD7* is a target gene induced by TGFβ that encodes for a negative regulator of the TGFβ/SMAD signaling pathway by associating with activated TβRI, thereby blocking the interaction, phosphorylation, and activation of SMAD2. Subsequent steps in SMAD signaling, including SMAD2–SMAD4 complex formation and its translocation to the nucleus are also inhibited by SMAD7.^57–59^ SMAD7 can also antagonize TGFβ signaling in the nucleus by disrupting the formation of the functional SMAD–DNA complex.^60,61^ Moreover, SMAD7 recruits E3 ubiquitin ligases such as SMAD ubiquitination regulatory factor 1/2 (SMURF1/2), WW domain-containing protein 1 (WWP1), and neural precursor cell-expressed developmentally downregulated 4-2 (NEDD4-2) to TβRI, and thereby promotes its ubiquitination-mediated proteasomal and/or lysosomal degradation.^62–64^ Naturally, the ubiquitination of TβRI can be reversed by a set of deubiquitinating enzymes, including ubiquitin-specific protease 4 (USP4), USP11, USP15, and ubiquitin carboxyl-terminal hydrolase L1.^65–68^

The target genes of the canonical TGFβ pathway that play key roles in regulating tumor progression are summarized in Fig. 3b.^69^ In the early stage of tumorigenesis, TGFβ promotes the expression of cyclin-dependent kinase inhibitors, including *p15*, *p21*, *p57*, and eukaryotic translation initiation factor 4E-binding protein 1 (*4E-BP1*), to induce cell cycle arrest.^70–74^ TGFβ represses the expression of genes encoding several growth-promoting factors, such as cell division cycle 25a (*CDC25A*), E2F transcription factor 1 (*E2F1*) and *C-MYC* proto-oncogene, to induce an anti-mitogenic cellular response.^75–79^ Inhibitor of DNA binding 1 (ID1) protein functions as a cell differentiation inhibitor and stimulator of cell proliferation, and long-term stimulation of cells by TGFβ can silence the *ID1* promoter and thereby drive epithelial cells to enter into a cytostatic program.^80^ ID1 was also shown to mediate the escape of pancreatic cancer from TGFβ tumor suppression.^81^ TGFβ can increase the expression of B-cell lymphoma 2 (*BCL2*)-interacting mediator of cell death (*BIM*), *BCL2*-modifying factor (*BMF*), *FAS* and death-associated protein kinase (*DAPK*) genes^82–85^ and decrease the expression of *BCL2* and *BCL-*extra large (*BCL-XL*) genes to induce cell apoptosis.^86,87^ In addition, TGFβ can suppress inflammation in the early stage of tumorigenesis by downregulating the expression of GATA-binding protein 3 (*GATA3*) and transcription factor T-box expressed in T cells (*T-BET*) genes to inhibit T cell differentiation.^88,89^ In the malignant stage, tumor cells can become non-responsive to the TGFβ-induced cytostatic and proapoptotic effects when genes that encode key components of the TGFβ pathway are mutated, and this results in the functional inactivation of their corresponding gene products. For example, *TβRII* mutations are frequently found in colorectal cancer (CRC),^90^ *SMAD2* mutations have been reported in CRC, hepatocellular carcinoma (HCC), and lung cancer,^91,92^ and the deletion or mutation of *SMAD4* is often identified in pancreatic cancer, CRC, and HCC.^93,94^ There are several advanced tumors that still retain an intact canonical TGFβ pathway, such as glioma, melanoma, and breast cancer. These cancer cells can avert TGFβ-induced cytostatic functions through the acquisition of activating mutations in oncogenes, for example, rat sarcoma (*RAS*) and *MYC*, and inactivating mutations in tumor suppressor genes, for example, *p53* and retinoblastoma protein (*Rb*). TGFβ can increase forkhead box P3 (*FOXP3)* gene expression,^95^ and decrease interferon-γ (*IFNγ*), MHC class I-chain-related molecules A (*MICA*), *granzyme A/B*, natural killer group 2 member D (*NKG2D*), natural cytotoxicity receptor 3 (*NKP30*), and *Perforin* gene expression to suppress immune function.^96–99^ Besides, TGFβ upregulates interleukin-11 (*IL11*), parathyroid hormone-related peptide (*PTHrP*), and *MMP9* to facilitate metastasis to specific organs.^100–102^ Moreover, TGFβ can promote angiogenesis by upregulating connective tissue growth factor (*CTGF*), vascular endothelial growth factor (*VEGF*), and *MMP2.*^103,104^

In addition to the canonical SMAD pathway, TGFβ can also initiate multiple noncanonical signaling pathways downstream of TGFβ receptors (Fig. 3c).^105^ For example, TβRI activates RHO small GTPases and then regulates the activity of RHO-associated protein kinase and LIM kinase to phosphorylate cofilin, leading to actin cytoskeleton reorganization that regulates cell adhesion, motility, and growth.^106^ TβRII can directly phosphorylate the cell polarity regulator PAR6 that regulates tight junctions and cell migration.^107^ TGFβ also induces the activation of TGFβ-activated kinase 1 (TAK1) to stimulate c-Jun NH2-terminal kinase (JNK) and p38 mitogen-activated protein kinase (MAPK) and nuclear factor κ-light-chain enhancer of activated B cells (NF-κB) pathways. These responses are mediated by tumor necrosis factor-associated factor 4 (TRAF4) and TRAF6 that interact with TGFβ receptors.^108–110^ Serine/threonine kinase protein kinase B (AKT) signaling can also be activated by TGFβ in a phosphatidylinositol 3-kinase (PI3K)-dependent manner.^111^ Besides, TGFβ can induce the phosphorylation of Src homology domain 2-containing protein (SHC) and then recruit growth factor receptor-binding protein 2 (GRB2) and son of sevenless (SOS) to activate the extracellular signal-regulated kinase (ERK) pathway through the RAS, RAF, and mitogen-activated protein kinase (MEK) pathways.^112^ A recent study discovered that the RAS-responsive element-binding protein 1 (RREB1) provides a molecular link between RAS and TGFβ pathways for coordinated induction of a developmental and fibrogenic EMT pathway.^113^ These non-SMAD signaling pathways are not unique to TGFβ; some are tightly regulated by receptor tyrosine kinases (RTKs) and crosstalk with the canonical SMAD signaling pathway.^114^ Moreover, TGFβ can also indirectly activate these pathways by inducing the expression of secreted growth factors, for example, platelet-derived growth factor (PDGF), which then acts in an autocrine or paracrine manner via specific receptors endowed with intrinsic tyrosine kinase activity.^115–117^

**Fig. 4:**
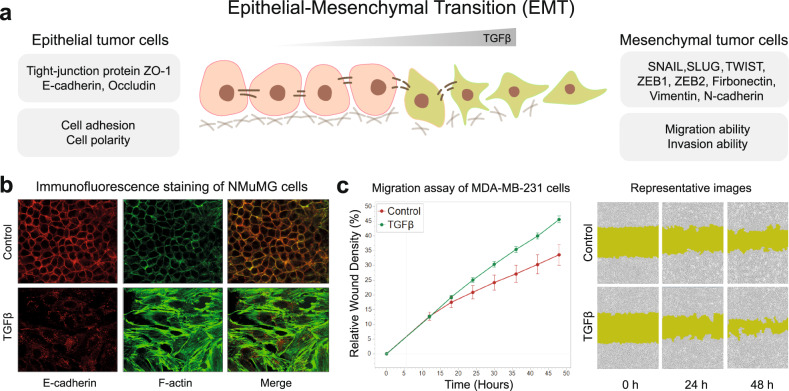
The functions of TGFβ upon the epithelial–mesenchymal transition (EMT). **a** Schematic of TGFβ mediation of the EMT process. **b** TGFβ promotes the EMT in non-transformed NAMRU mouse mammary gland (NMuMG) epithelial cells, as visualized by immunofluorescent staining of cells with anti-E-cadherin antibody (red) and phalloidin (green) to measure filamentous actin expression in the absence and after treatment with 5 ng/ml TGFβ3 for 48 h. The typical morphological change from epithelial- to fibroblast-like cells, decreased E-cadherin, and accumulated striated fibers are observed in the NMuMG cells upon TGFβ stimulation. **c** TGFβ promotes the migration of MDA-MB-231 human breast cancer cells, as determined via real-time imaging of a wound-healing scratch assay. Left, graph showing the time-lapse relative migration rate; right, images of the cells taken when the initial scratch was made (0 h) and after 24 and 48 h in the absence or presence of 5 ng/ml TGFβ3

Furthermore, TGFβ plays an important role in inducing the metastatic capacity of tumor cells by promoting the EMT through the upregulation of transcription factors such as *SNAIL*, *SLUG*, *TWIST*, and *ZEB1/2* through either SMAD- or non-SMAD-dependent pathways.^118–124^ Accompanying the increase of TGFβ levels, EMT leads epithelial tumor cells to lose the capability of adhesion, polarity, and tight junctions by decreasing levels of tight-junction proteins zona occludence-1 (ZO-1), E-cadherin, and occludin, and acquire a highly migratory and invasive mesenchymal phenotype by the increased levels of fibronectin, vimentin, and N-cadherin (Fig. 4a).^19^ EMT has diverse phenotypic manifestations with intermediate epithelial/mesenchymal states and is a reversible process. This has been referred to as epithelial cell plasticity (EMP).^125^ TGFβ can induce different states of EMP and the cellular response to TGFβ is context-dependent; inflammatory factors, Wnt, Notch, Hippo, and Hedgehog interplay with TGFβ to orchestrate the EMP response.^126^ In Fig. 4b, we show that TGFβ promotes the EMT in non-transformed mouse mammary gland (NMuMG) epithelial cells.^127^ The NMuMG cell line is a frequently used model system to investigate TGFβ-induced EMT.^128,129^ The ability of TGFβ to promote the migration of the human mesenchymal triple-negative breast cancer (TNBC) MDA-MB-231 cells is shown in Fig. 4c.^130^ MDA-MB-231 is highly aggressive and its metastasis to bone occurs in a TGFβ/SMAD-dependent manner.^101,119,131^

## Functions of TGFβ in the TME

**Fig. 5:**
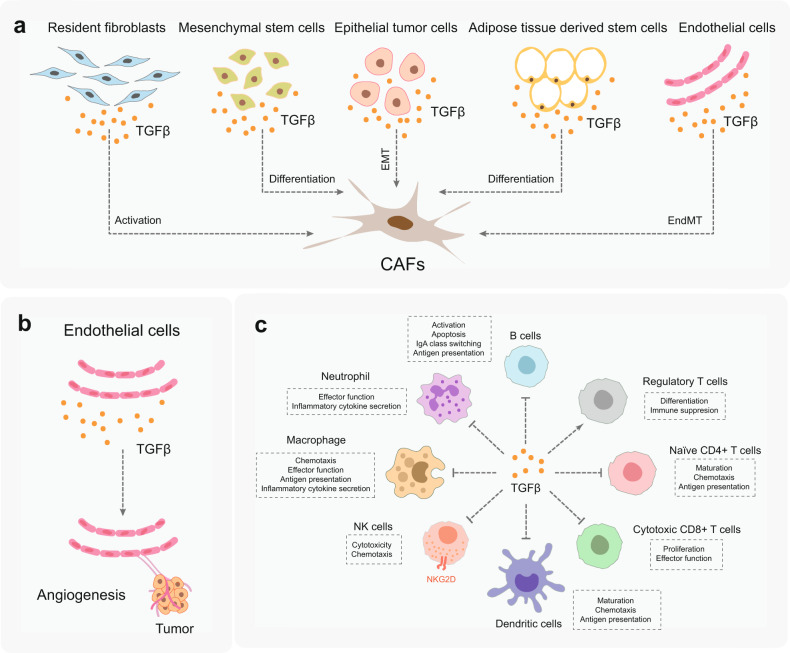
Functions of TGFβ in the tumor microenvironment (TME). **a** TGFβ can activate/differentiate resident fibroblasts, mesenchymal stem cells, epithelial tumor cells, adipose tissue-derived stem cells, and endothelial cells into cancer-associated fibroblasts (CAFs) in the TME. **b** TGFβ promotes angiogenesis in the TME by acting directly and indirectly on endothelial cells stimulating their proliferation, migration, and sprouting. **c** TGFβ crucially suppresses the immune system by regulating the functions of immune cell populations in the TME. The specific actions of TGFβ are indicated in the boxes next to the different immune cells that are depicted

The TME consists of ECM, cytokines, and a large population of different cell types, including resident and infiltrating CAFs, immune-related cells, endothelial cells, and adipocytes that surround the tumor cells. The multipronged effects of TGFβ on tumor stroma cells built the TME, including its capacity to stimulate ECM production, activate CAFs, suppress the immune system, and promote angiogenesis. Notably, tumor stromal cells are also the main sources of inflammatory factors, including TGFβ. Reciprocally, the TME exerts profound effects on tumor growth and progression. In this section, we summarize the functions of TGFβ in the TME (Fig. 5).

### TGFβ and CAFs in the TME

CAFs are key components of the TME and play roles in providing a favorable environment to support tumor progression by producing ECM and cytokines, stimulating immune evasion, and promoting angiogenesis.^132–134^ Next to tumor-promoting CAFs, also tumor-suppressive CAFs have been identified.^135^ Within a tumor, the CAF population can be highly heterogeneous. For example in breast tumors, at least four distinct CAF subpopulations, that is, vascular CAFs, matrix CAFs, cycling CAFs, and developmental CAFs (dCAFs), were defined.^136^ In pancreatic tumors, CAFs have been classified into inflammatory CAFs, which are found distant from tumor foci with high IL6 and low α-smooth muscle actin (α-SMA) expression, and myofibroblastic CAFs that are located adjacent to tumor foci with high α-SMA expression and that demonstrate a strong TGFβ responsiveness.^137–139^ The characterization of molecular markers and mechanistic insights of different CAF subtypes may provide opportunities for their precise targeting in cancer therapy.^140^

During the formation of CAFs, TGFβ is a major driver in activating resident quiescent fibroblasts, and differentiating bone marrow-derived mesenchymal stem cells and adipose tissue-derived stem cells into CAFs (Fig. 5a).^141–144^ The activation of the TGFβ signaling pathway can also induce epithelial cancer cells into myofibroblasts via EMT, and differentiate endothelial cells into fibroblast-like cells by endothelial-mesenchymal transition (EndMT) (Fig. 5a).^145,146^ In turn, CAFs autocrine TGFβ can serve as an attractant to recruit more fibroblasts into the TME around the tumor invasion front and promote metastasis.^147,148^ CAFs surround and throughout the tumor often restrict the accessibility of anti-cancer drugs to tumor cells since the dense CAFs with stiff surrounding matrix can reduce the density of blood vessels and form a physical barrier surrounding the tumor cells.^149^ In hypoxic TME, CAF-secreted TGFβ2 can cooperate with hypoxia-inducible factor to increase stemness phenotype and induce robust chemotherapy of cancer cells.^150^ Radiation-induced TGFβ signaling can activate CAFs in the TME, which enhances the invasiveness of the associated tumor cells via promoting EMT.^151^ One recent study in which *TβRII*-mutant pancreatic cancer was investigated, found that the anti-tumor efficacy of TβRII blockade is due to the inhibition of the TGFβ signaling in CAFs.^152^ Furthermore, an increasing number of studies reported that the overactive TGFβ signaling in CAFs is a major reason of immunotherapy failure.^153^ Analysis of the single-cell landscape of CAFs in pancreatic cancer identified a TGFβ-driven CAF population expressing leucine-rich repeat-containing 15 (LRRC15).^154^ This transmembrane protein is associated with poor outcome in immunotherapy trial data.^154^ It will be interesting to explore if the targeting of LRRC15^+^ CAF in the TME will boost the response of cancer patients towards immune checkpoint blockade therapy.^154^

### TGFβ and endothelial cells in the TME

Endothelial cells are located at the surface of blood and lymphatic vessels. Blood vessels nourish tumors by delivering blood/oxygen/nutrients, removing waste products, and mediating the entry and exit of immune cells and other substances.^155–158^ A positive correlation between the TGFβ level and microvessel density was observed in various types of tumors.^159^ Endothelial cells express two TβRIs (activin receptor-like kinase 1 (ALK1) and TβRI/ALK5). The TGFβ–ALK5 signaling axis can directly mediate inhibition of endothelial cell proliferation and migration (Fig. 5b).^160,161^ Indirectly, the expression of some key angiogenic factors, such as VEGF, CTGF, and fibroblast growth factor 2 (FGF2), can be induced by the TGFβ–ALK5 signaling axis.^162–165^ In the presence of endoglin, the TGFβ-ALK1 signaling axis can directly promote endothelial cell proliferation and migration.^160,166^ Moreover, the JNK pathway also mediates the proangiogenic response of TGFβ in endothelial cells.^167^ A recent study showed that TGFβ can promote VEGF-C production in tubular epithelial cells, macrophages, and mesothelial cells to induce lymphangiogenesis in renal and peritoneal fibrosis. However, the function of TGFβ in the formation of the cancer-associated lymphatic system is not well understood.^168^

### TGFβ and immune-related cells in the TME

There are a variety of innate and adaptive immune cells dispersed throughout the TME (Fig. 5c). During tumorigenesis, myeloid cells (including myeloid-derived suppressor cells (MDSCs), macrophages, neutrophils) typically accumulate in the early stage of tumor outgrowth to suppress the T cell response and sustain an immunosuppressive environment.^169^ Dendritic cells (DCs) deliver tumor antigens to T cells and natural killer (NK) cells that exert antitumor cytotoxic effects.^170^ However, antitumor immune reactions often become suppressed during tumor development; TGFβ can exhibit pivotal immunosuppressive effects on the intrinsic antitumor potential of DCs and NK cells in the TME (Fig. 5c). At an early stage of cancer, TGFβ mitigates myeloid proliferation and differentiation by repressing the expression of cytokine IFNγ.^171,172^ Progressively, myeloid cells in advanced-stage cancer produce TGFβ and MMPs that further inhibit antitumor immune reactions and promote tumor metastasis.^173–175^ Indeed, experimentally induced inactivation of TGFβ signaling in myeloid cells can lead to an increase in antitumor activity.^175,176^ TGFβ blocks the activation of NK cells and suppresses their cytotoxic potential by inhibiting C-type lectin receptor NKG2D expression directly and indirectly.^177^ IL1 receptor-associated kinase M (IRAK-M) is a potent negative regulator of Toll-like receptor (TLR) signaling and is predominantly expressed in macrophages.^178^ By inducing IRAK-M expression and antagonizing TLR signaling, TGFβ protects tumors from the potential TLR-mediated antitumor activities of macrophages.^179^

TGFβ suppresses adaptive immunity during cancer progression mainly by inhibiting the activation, proliferation, differentiation, and migration of T cells. TGFβ can suppress the differentiation of naive CD4^+^ helper T cells into distinct effector subtypes. However, it induces the conversion of naive T cells into T_reg_ cells (previously known as suppressor T cells) that suppress the immune response.^180^ A recent study showed that depletion of TβRII in CD4^+^ T cells suppresses cancer progression as a result of tissue healing and remodeling of the blood vasculature, causing cancer hypoxia and death in distant avascular regions.^181^ TGFβ can block the activation and maturation of cytotoxic CD8^+^ T cells by repressing the tumor antigen processing and presentation of DCs and inhibit CD8^+^ T cell proliferation through suppressing the expression of IFNγ and IL2.^172,182–184^ TGFβ promotes antigen-induced programmed cell death protein-1 (PD-1) expression in CD8^+^ T cells, which causes T cell exhaustion.^185^ Recently, researchers found that the TGFβ signal maintains the immune-suppressive identity of CD8^+^ T_reg_ cells. Transcription factor Eomesodermin (Eomes) controls the follicular location of CD8^+^ T_reg_ cells.^186^ Both TGFβ and Eomes coordinate to promote the homeostasis of CD8^+^ T_reg_ cells.^186^ TGFβ can regulate the activation, proliferation, apoptosis of B cells, and stimulate the antibody switching in B cells. However, its function on B cell-mediated antitumor immunity is not well investigated.^187–189^

## Pharmacological interventions of TGFβ in cancer therapy

**Fig. 6:**
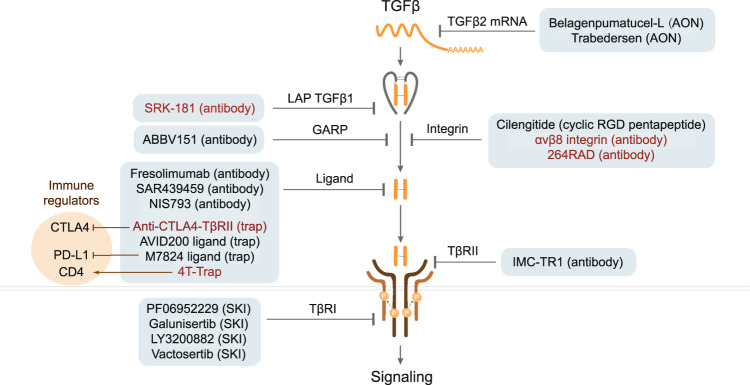
Schematic of strategies utilized in (pre)clinical trials targeting TGFβ signal transduction for cancer therapy. Various pharmacological interventions are grouped into the targeting of different TGFβ signaling components, that is, TGFβ mRNA, GARP/integrins that are involved in activation of latent TGFβ, and ligands that interact with TGFβ receptors and TβRI kinase activity. The promising new targeting molecules that have been studied in pre-clinical models are highlighted in red color. Different strategies for targeting TGFβ signaling, including antisense oligonucleotide (AON), neutralizing antibody (antibody), cyclic RGD pentapeptide, TGFβ ligand trap (trap), and small-molecule kinase inhibitor (SKI) are indicated. The immune regulatory targets (CTLA4/PD-L1/CD4) of the bispecific molecules that sequester TGFβ with a TβRII extracellular domain containing trap are highlighted in the orange circle

There are numerous anti-cancer pharmacological interventions that target specific mediators of TGFβ signaling pathway or TGFβ activators, which have been tested in human clinical trials or that displayed very promising results in pre-clinical animal models (Fig. 6). In this section, we introduce the recent advancements and bottlenecks of the main anti-TGFβ strategies, including neutralizing antibodies, ligand traps, small-molecule kinase inhibitors, and antisense oligonucleotides (AONs), and summarize the pharmacological interventions that have been or are currently being studied in clinical trials (Table 1).

**Table 1:** Summary of pharmacological strategies targeting TGFβ for cancer therapy in clinical trials (information was obtained from https://www.clinicaltrials.gov/)

Strategy	Drug	Target	Cancer	Identifier	Phase	Treatment	Status
Neutralizing antibody	Fresolimumab	TGFβ1/2/3	Glioma	NCT01472731	2	Monotherapy	Completed
			Metastatic breast cancer	NCT01401062	2	Combination with radiotherapy	Completed
			Relapsed malignant pleural mesothelioma	NCT01112293	2	Monotherapy	Completed
			Renal cell carcinoma or malignant melanoma	NCT00356460	1	Monotherapy	Completed
			Early stage non-small cell lung cancer	NCT02581787	2	Combination with radiotherapy	Recruiting
	SAR439459	TGFβ1/2/3	Advanced solid tumors	NCT03192345	1	Monotherapy/combination with cemiplimab	Recruiting
	NIS793	TGFβ1/2/3	Metastatic pancreatic ductal adenocarcinoma	NCT04390763	2	Monotherapy/combination with spartalizumab/gemcitabine and paclitaxel	Active
			Breast/lung/hepatocellular/colorectal/pancreatic/renal cancer	NCT02947165	1	Monotherapy/combination with spartalizumab	Recruiting
	IMC-TR1	TβRII	Advanced solid tumor	NCT01646203	1	Monotherapy	Completed
	ABBV151	GARP:TGFβ1	Advanced/metastatic solid tumors	NCT03821935	1	Monotherapy/combination with ABBV-181	Recruiting
Ligand trap	AVID200	TGFβ1/3	Advanced/metastatic solid tumors	NCT03834662	1	Monotherapy	Active
	M7824	TGFβ/PD-L1	Advanced non-small cell lung cancer	NCT03631706	3	Monotherapy	Recruiting
			HPV-associated cancers	NCT04432597	2	Combination with HPV vaccine PRGN-2009	Recruiting
			Cholangiocarcinoma/gallbladder cancer	NCT03833661	2	Monotherapy	Active
			Recurrent respiratory papillomatosis	NCT03707587	2	Monotherapy	Active
			Biliary tract cancer	NCT04066491	3	Combination with gemcitabine and cisplatin	Recruiting
			Relapsed small cell lung cancers	NCT03554473	2	Combination with topotecan/temozolomide	Recruiting
			Metastatic triple-negative breast cancer	NCT03579472	1	Combination with eribulin mesylate	Recruiting
			Thymoma/thymic carcinoma	NCT04417660	2	Monotherapy	Active
			Stage II–III HER2-positive breast cancer	NCT03620201	1	Monotherapy	Recruiting
			Advanced Kaposi sarcoma	NCT04303117	2	Combination with M9241	Recruiting
			Advanced adenocarcinoma of the pancreas	NCT03451773	2	Combination with gemcitabine	Completed
			Metastatic colorectal cancer/advanced solid tumors	NCT03436563	2	Monotherapy	Recruiting
			Advanced pancreas cancer	NCT04327986	2	Combination with M9241 and radiotherapy	Active
			Metastatic non-prostate genitourinary malignancies	NCT04235777	1	Combination with M9241/radiotherapy	Recruiting
			Resectable head and neck squamous cell carcinoma	NCT04247282	2	Monotherapy/combination with N-803 and TriAd Vaccine	Recruiting
			Metastatic/locally advanced urothelial cancer	NCT04349280	1	Monotherapy	Active
			HER2-negative breast cancer	NCT03524170	1	Combination with radiotherapy	Recruiting
			Advanced-stage breast cancer	NCT04296942	1	Combination with ado-trastuzumab emtansine, entinostat, and BN-brachyury vaccine	Recruiting
			AT-hook 2 (HMGA2) expressing triple-negative breast cancer	NCT04489940	2	Monotherapy	Active
			Platinum-experienced cervical cancer	NCT04246489	2	Monotherapy	Recruiting
			Recurrent/second primary head and neck squamous cell cancer	NCT04220775	2	Combination with radiotherapy	Recruiting
			Advanced small bowel/colorectal cancers	NCT04491955	2	Combination with N-803, M9241, and CV301 vaccine	Active
Small-molecule inhibitors	Galunisertib	TβRI	Advanced hepatocellular carcinoma	NCT02906397	1	Combination with radiotherapy	Active
			Metastatic castration-resistant prostate cancer	NCT02452008	2	Combination enzalutamide	Recruiting
			Rectal cancer	NCT02688712	2	Combination fluorouracil/capecitabine and radiotherapy	Recruiting
			Metastatic breast cancer	NCT02538471	2	Combination with radiotherapy	Terminated
			Metastatic pancreatic cancer	NCT02734160	1	Combination with durvalumab	Terminated
			Advanced refractory solid tumors	NCT02423343	1	Combination with nivolumab	Active
			Recurrent/refractory non-small cell lung cancer	NCT02423343	2	Combination with nivolumab	Active
			Hepatocellular carcinoma	NCT01246986	2	Combination with sorafenib/ramucirumab	Active
			Metastatic androgen receptor-negative triple-negative breast cancer	NCT02672475	1	Combination with Paclitaxel	Recruiting
			Newly diagnosed malignant glioma	NCT01220271	2	Combination with temozolomide and radiotherapy	Completed
			Carcinosarcoma of the uterus or ovary	NCT03206177	1	Combination with paclitaxel/carboplatin	Recruiting
			Advanced/metastatic unresectable pancreatic cancer	NCT01373164	2	Combination gemcitabine/placebo	Completed
	Vactosertib	TβRI	Advanced solid tumors	NCT02160106	1	Monotherapy	Completed
			Refractory multiple myeloma	NCT03143985	1	Combination with pomalidomide	Recruiting
			Urothelial carcinoma	NCT04064190	2	Combination with durvalumab	Active
			Metastatic colorectal cancer	NCT03724851	1	Combination with pembrolizumab	Recruiting
			Advanced non-small cell lung cancer	NCT03732274	2	Combination with durvalumab	Recruiting
			Myeloproliferative neoplasm	NCT04103645	2	Monotherapy	Active
			Advanced desmoid tumor	NCT03802084	2	Combination with imatinib	Recruiting
			Metastatic gastric cancer	NCT03698825	2	Combination with paclitaxel	Recruiting
			Metastatic pancreatic ductal adenocarcinoma	NCT04258072	1	Combination with irinotecan, fluorouracil, and leucovorin	Active
	LY3200882	TβRI	Solid tumors	NCT02937272	1	Monotherapy	Active
			Activated colorectal cancer	NCT04031872	2	Combination with capecitabine	Active
	PF06952229	TβRI	Advanced solid tumors	NCT03685591	1	Monotherapy	Recruiting
	Cilengitide	αvβ3/5	Diffuse intrinsic pontine glioma	NCT01165333	1	Combination with radiotherapy	Completed
			Advanced non-small cell lung cancer	NCT00842712	2	Combination with cetuximab and platinum-based chemotherapy	Completed
			Brain metastases from lung cancer	NCT00884598	1	Combination with radiotherapy	Completed
			Advanced solid tumors/glioblastoma multiforme	NCT01122888	1	Combination with sunitinib	Terminated
			Metastatic prostate cancer	NCT00103337	2	Monotherapy	Completed
			Recurrent glioblastoma multiforme	NCT00093964	2	Monotherapy	Completed
			Diagnosed inoperable glioblastoma	NCT01558687	1	Monotherapy	Terminated
			Advanced solid tumors/lymphoma	NCT00077155	1	Monotherapy	Completed
			Childhood recurrent/progressive high-grade glioma	NCT00679354	2	Monotherapy	Completed
			Newly diagnosed glioblastoma	NCT00689221	3	Combination temozolomide and radiotherapy	Completed
			Childhood refractory primary brain tumors	NCT00063973	1	Monotherapy	Completed
			Prostate cancer	NCT00121238	2	Monotherapy	Completed
			Unresectable/metastatic melanoma	NCT00082875	2	Monotherapy	Terminated
			Acute myeloid leukemia	NCT00089388	2	Monotherapy	Terminated
			Advanced solid tumors	NCT00022113	1	Monotherapy	Completed
			Undergoing surgery for recurrent/progressive glioblastoma multiforme	NCT00112866	2	Monotherapy	Terminated
			HIV-related Kaposi’s sarcoma	NCT00006222	1	Monotherapy	Terminated
			Locally advanced/metastatic cancer	NCT00004258	1	Monotherapy	Completed
			Progressive/recurrent glioma	NCT00006093	2	Monotherapy	Completed
			Relapsed/refractory high-grade gliomas/diffuse intrinsic pontine gliomas	NCT01517776	2	Combination with temozolomide and radiotherapy	Terminated
			Recurrent/metastatic squamous cell carcinoma of the head and neck	NCT00705016	2	Combination with cetuximab, cisplatin, and fluorouracil	Completed
			Triple-negative breast cancer/advanced solid tumors that cannot be removed by surgery	NCT01276496	1	Combination with paclitaxel	Completed
			Newly diagnosed MGMT-promoter unmethylated glioblastoma	NCT01044225	3	Combination with radiotherapy and temozolomide	Terminated
Antisense oligonucleotide	Trabedersen	TGFβ2	Advanced tumors known to overproduce TGFβ2	NCT00844064	1	Monotherapy	Completed
			Glioblastoma and anaplastic astrocytoma	NCT00761280	3	Monotherapy	Terminated
			Recurrent/refractory high-grade glioma	NCT00431561	2	Monotherapy	Completed
	Lucanix	TGFβ2	Non-small cell lung cancer	NCT01058785	2	Monotherapy	Completed
			Non-small cell lung cancer	NCT00676507	3	Monotherapy	Completed

### Neutralizing antibodies

Neutralizing antibodies can be selectively directed against critical domains of native ligands and extracellular domains of receptors to block their biological activity. Fresolimumab (GC1008), a human IgG4κ monoclonal antibody that neutralizes TGFβ1, 2, and 3, demonstrated acceptable safety and antitumor activity in a phase 1 clinical trial for patients with malignant melanoma or renal carcinoma.^190^ Phase 2 clinical trials of this antibody have been conducted for patients with glioma, metastatic breast cancer, or relapsed malignant pleural mesothelioma, which showed good toleration.^191–193^ The phase 2 clinical evaluation of this antibody for the treatment of early-stage non-small cell lung cancer (NSCLC) is still ongoing (NCT02581787). LY3022859 is an anti-TβRII IgG1 monoclonal antibody blocking TGFβ binding to the ectodomain of TβRII that showed significant antitumor function against primary tumor growth and metastasis in several mice tumor models.^194^ When tested for use in the treatment of advanced solid tumors in a phase 1 clinical trial, the maximum-tolerated dose for this antibody was not determined since the patients suffered from uncontrolled cytokine release despite prophylaxis.^195^

Integrins are major activators of TGFβ ligand, including αvβ1, αvβ3, αvβ5, αvβ6, and αvβ8.^46,196–199^ An increase number of studies showed that integrin-induced TGFβ signaling pathway modulates the tumor stroma and in particular the immune microenvironment.^200^ Thus, besides their role to promote cell proliferation, survival, and migration, the ability of αv integrins to stimulate TGFβ activation contributes to cancer progression.^201,202^ Thus, integrins can be pharmacological targets for cancer treatment, and their targeting may enhance the efficacy of immune therapies.^203^ For example, previous research found that αvβ8 integrin-expressing tumor cells can evade host immunity by upregulating TGFβ signaling in immune cells.^204^ The engineered antibody against αvβ8 integrin can block the release of active TGFβ by cancer cells and promote the anti-tumor immune reaction in pre-clinical mice models of colon carcinoma and lung cancer.^205^ A recent study showed that αvβ6-neutralizing antibody 264RAD can suppress TGFβ signaling and significantly reduce tumor growth in αvβ6-positive human pancreatic ductal adenocarcinoma (PDAC) xenografts mice model and immunocompetent transgenic mice bearing αvβ6-positive PDAC tumors.^206^ Although most studies showed that anti-αvβ integrin therapy has promise in cancer treatment, an opposite finding was reported for antibody-mediated inhibition of αvβ6 that while leading to a strong decreased TGFβ signaling, it promoted pancreatic cancer progression.^207^

### Ligand traps

TGFβ ligand traps are TGFβ receptor ectodomain-based chimeric fusion proteins that are rationally designed to prevent TGFβs from binding to their receptors. AVID200 is a selective trap of TGFβ1 and TGFβ3 that showed the capacity to enhance the anti-tumor efficacy by inhibiting the growth of syngeneic 4T1 TNBC homograft in immunocompetent host mice.^187^ A phase I clinical trial of AVID200 revealed that it was safe and well tolerated for patients with advanced solid tumors.^208,209^

### Small-molecule inhibitors

Small-molecule kinase inhibitors of TβR kinases are undergoing intensively clinical trials for anti-TGFβ signaling in cancer treatment. Galunisertib (LY2157299) is an orally available small-molecule inhibitor that selectively binds to TβRI (and weakly to TβRII) and inhibits its kinase activity. It showed favorable preclinical results for antitumor growth, for example, in Calu6 and EMT6-LM2 mouse tumor models.^210^ In phase 1 clinical trials, this inhibitor exhibited acceptable safety and dose tolerability for patients with HCC/metastatic pancreatic cancer/malignant glioma/advanced solid tumors.^211–214^ Phase 1/2 clinical trials of this inhibitor for patients with metastatic prostate cancer/TNBC/advanced HCC/rectal cancer/recurrent glioblastoma are ongoing.^213,215,216^ A phase 2 study of this inhibitor for patients with advanced HCC showed acceptable safety and prolonged overall survival.^215^ However, in a phase 2 study of patients with recurrent glioblastoma, this inhibitor failed to improve the overall survival.^217^ Vactosertib (TEW-7197) is another orally bioavailable kinase inhibitor of TβRI that revealed promising antitumor function in a mouse model of myeloma.^218^ This inhibitor displayed a favorable safety profile and antitumor efficacy in phase 1 clinical trials for patients with advanced solid tumors or desmoid tumors.^219,220^ Phase 1 clinical trial investigation of this inhibitor for patients with refractory multiple myeloma/metastatic CRC/metastatic PDAC and phase 2 study for patients with metastatic gastric cancer/CRC/urothelial carcinoma/ NSCLC/myeloproliferative neoplasm/advanced desmoid tumors are ongoing.^221–224^ LY3200882 is a next-generation highly selective potent ATP-competitive TβRI inhibitor that showed antitumor efficacy in a preclinical mouse model of TNBC cancer.^225^ The phase 1 clinical trial results showed that LY3200882 had a tolerable safety profile and early signs of antitumor efficacy for patients with advanced or metastatic cancers (NCT04031872). PF06952229 is a selective and orally available TβRI inhibitor that is being studied in phase 1 clinical trials for patients with advanced/metastatic breast cancer/castration-resistant prostate cancer (NCT03685591).

Previous studies showed that genetically and pharmacologically inhibition of αvβ integrin can inhibit TGFβ signaling and suppress metastasis.^201,203^ For example, the cyclic RGD pentapeptide cilengitide (EMD121974) is a potent and selective inhibitor of integrin αvβ3/5 that showed a reduction of TGFβ1 and TGFβ2 messenger RNA (mRNA) and protein expression, SMAD2 phosphorylation, and TGFβ-mediated reporter gene activity in most glioma cells.^226^ The intracranial LN-308 glioma xenograft mice model also displayed decreased SMAD2 phosphorylation in response to cilengitide.^226^ This inhibitor has been evaluated in phase 1 clinical trials for patients with brain tumors/lung cancer/advanced solid tumor/lymphoma/HIV-related Kaposi’s sarcoma;^227–230^ in phase 2 clinical trials for patients with NSCLC/prostate cancer/metastatic melanoma/acute myeloid leukemia/head neck squamous cell carcinoma;^231–237^ and in phase 3 for patients with glioblastoma.^238^ The clinical trials of cilengitide were terminated for the treatment of patients with metastatic melanoma, acute myeloid leukemia, or HIV-related Kaposi’s sarcoma since it neither exhibited antitumor efficacy nor improved overall survival. However, the phase 3 clinical study of cilengitide in glioblastoma patients showed well toleration and single-agent activity.^237,238^

### Antisense oligonucleotides

AONs are short oligonucleotides designed to suppress the expression of specific genes by blocking their translation.^239^ Trabedersen (AP12009) is a phosphonothioate antisense oligodeoxynucleotide that specifically targets TGFβ2 mRNA.^240^ Trabedersen was evaluated in the phase 1 clinical studies in patients with melanoma/pancreatic cancer/CRC, and showed good safety and encouraging survival results.^241^ The phase 2 clinical trials’ evaluation of this AON for patients with glioblastoma or anaplastic astrocytoma enabled the determination of the optimal dose for further clinical development.^242^ However, the phase 3 clinical trial of trabedersen for patients with glioblastoma was terminated because of the lack of patient recruitment.^243–245^ Lucanix (belagenpumatucel-L) is a vaccine expressing AON TGFβ2 RNA that was evaluated in clinical trial studies for NSCLC patients.^246,247^ This vaccine did not meet its survival endpoint in a phase 3 clinical trial for NSCLC patients; however, it improved the overall survival of patients who had completed chemotherapy within the previous 12 weeks.^248^

## Targeting TGFβ in cancer therapy: challenges and opportunities

### Overcoming the adverse effects of anti-TGFβ therapies

Cancer patients who were treated with blockers of TGFβ signaling can experience side effects if the function of TGFβ in physiological processes is compromised. For example, some TβRI kinase inhibitors showed therapeutic effects in cancer patients, but their cardiac toxicity at high doses (hemorrhagic, degenerative, and inflammatory lesions in heart valves) and skin toxicity (eruptive keratoacanthomas, hyperkeratosis, cutaneous squamous-cell carcinomas, and basal cell carcinoma) limits their safe therapy window.^22^ These adverse effects have (and continue to) challenge the clinical application of many other anti-TGFβ therapies. Careful dosing of TGFβ inhibitors to cancer patients may attenuate toxicity. Moreover, the adverse effects may be mitigated by pulsatile therapy, in which the patient has so-called “drug holidays” during the dosing period. Galunisertib (LY2157299) has been applied as a therapy regimen of 2 weeks on and 2 weeks off drug treatment to reduce the (cardiac) side effects.^249^ In addition, predictive biomarkers may aid in selecting patients who benefit most from treatment with anti-TGFβ agents. Transcriptional profiling of samples from patients with many different cancer types, including glioblastoma, pancreatic cancer, breast cancer, ovarian cancer, CRC, and NSCLC, revealed that cancer patients with mesenchymal subtypes have high expression of TGFβ target genes and that this correlates with poor prognosis. Therefore, subgroups of patients with cancers that carry a mesenchymal phenotype may particularly benefit from anti-TGFβ therapies.^250–255^

### Perspectives on the synergy of combination therapies

**Fig. 7:**
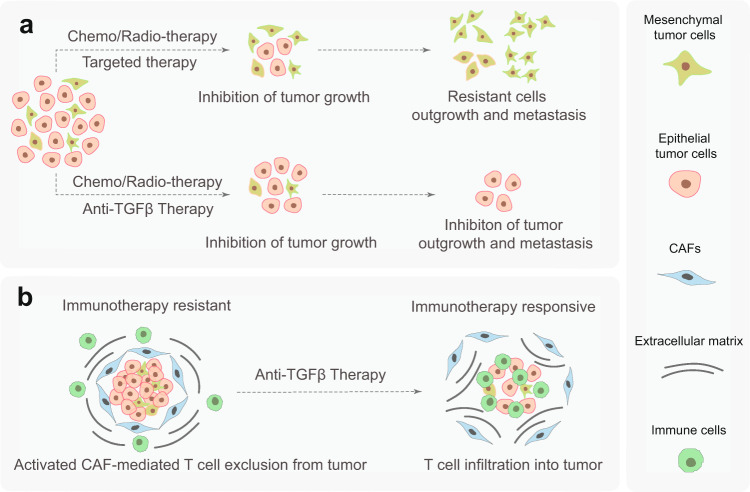
Sketch of synergistic combination therapies. **a** Chemo/radio/targeted therapy alone inhibits the growth of epithelial-like tumor cells, and in combination with anti-TGFβ therapy, invasive escape and resistance to these therapies are attenuated, and metastasis of mesenchymal tumor cells is restrained. **b** Activated CAFs mediated by high TGFβ activity suppresses immunotherapy efficacy by blocking T cell infiltration into tumors and inducing T cell dysfunction. In combination with anti-TGFβ therapy; however, T cell exclusion is inhibited, and the antitumor efficacy of the immunotherapy is improved

Increased TGFβ activity has been implicated in the resistance to various anticancer therapies, including cytotoxic chemotherapy, targeted therapy, radiotherapy, and immunotherapy.^4^ Therefore, combining anti-TGFβ therapy with these established strategies may dampen therapy resistance (Fig. 7). A future opportunity and challenge are to identify the optimal combination of a synergistic therapy regimen for each individual patient.

#### Anti-TGFβ therapy with chemotherapy, targeted therapy, or radiotherapy

Chemotherapy is a frequent standard first-line cancer therapy regimen consisting of one or more anticancer chemicals designed to stop and kill rapidly proliferating cancer cells. However, chemotherapy has strong side effects due to its toxicity to normal cells. Targeted therapy is performed by using small molecules that interfere with specific signaling pathways that promote cancer cell proliferation and survival.^256^ Examples of such small-molecule compounds are selective tyrosine kinase inhibitors.^257^ Selective small-molecule inhibitors have less severe side effects than standard chemotherapy, but both types of therapy can lead to therapy resistance and relapse, as some cells within the heterogeneous tumor population survive and proliferate after an initial beneficial anticancer response.^258^ TGFβ-induced cancer cell acquisition of a mesenchymal phenotype has emerged as a key mechanism that confers the therapy-resistant and stemness phenotype to cancer cells.^22^ Blocking TGFβ may kill two birds with one stone by impairing metastasis and preventing resistance against anticancer treatments.

TβRI kinase inhibitors, such as galunisertib (LY2157299), have been combined with multiple chemotherapies, including paclitaxel, sorafenib, or gemcitabine in patients with TNBC, glioblastoma, HCC, or PDAC to improve antitumor efficacy.^215,216,259–261^ LY3200882 is combined with gemcitabine/paclitaxel/cisplatin in an ongoing phase 1 trial for the treatment of patients with solid tumors (NCT02937272), or is tested in combination with capecitabine for patients with resistant metastatic CRC (NCT04031872). The efficacy of a combination of vactosertib (TEW-7197) and paclitaxel is explored in an ongoing phase 1/2 clinical trial for the treatment of metastatic gastric cancer (NCT03698825). NIS793 is a pan anti-TGFβ-neutralizing antibody that is currently being tested in a phase 2 clinical trial for patients with metastatic PDAC in combination with gemcitabine/nab-paclitaxel chemotherapy (NCT04390763).^262^ TβRI kinase inhibitors have also been combined with targeted therapies to overcome drug resistance in pre-clinical cancer models. For example, downregulation of mediator complex subunit 12 (MED12) leads to resistance to multiple tyrosine kinase inhibitors in multiple cancer cells. Mechanistically, MED12 depletion was found to increase TβRII protein levels, mediate MEK/ERK activation, and induce the expression of mesenchymal markers. Treatment with the TβRI kinase inhibitor galunisertib (LY2157299) sensitized the therapeutic response of metastatic non-small cell lung cancers with downregulated MED12 to RTK inhibitors.^263^

In radiotherapy, ionizing radiation is used to kill or control the growth of malignant cells. Some types of cancer are notably radioresistant, such as glioblastoma, which produces abundant TGFβ in the microenvironment that leads to the radioresistance of glioma-initiating cells.^264^ Radiation can also cause normal tissues to become more fibrotic by inducing the expression and release of active TGFβ.^265^ Therefore, the combination of anti-TGFβ therapy with radiotherapy may improve treatment by enhancing tumor cell radiosensitivity and protecting normal tissues.^266,267^ In a phase 2 clinical trial, the TGFβ-blocking antibody fresolimumab (GC1008) was combined with focal irradiation for the treatment of metastatic breast cancer, and it was found to prolong median overall survival and demonstrated a favorable systemic immune response.^268,269^ In addition, fresolimumab (GC1008) combined with stereotactic ablative radiotherapy is in a phase 2 clinical trial for early-stage NSCLC treatment (NCT02581787). The combination of the TβRI kinase inhibitor LY3200882 with intensity-modulated radiotherapy is currently being tested in a phase 1 clinical trial of patients with solid tumors (NCT02937272).

#### Anti-TGFβ therapy with immunotherapy

Immune checkpoints are crucial regulators in the process of the immune response. Two types of immune checkpoint signals have been described. Costimulatory signals are mediated by CD27, CD28, CD40, CD134, CD137, and so on, for cytotoxic effector T cell proliferation and their migration toward the tumor.^270,271^ Coinhibitory signals increase T cell exhaustion and dysfunction, and this process engages coinhibitory molecules, such as PD-1, cytotoxic T-lymphocyte-associated protein 4 (CTLA4), lymphocyte activation gene 3 (LAG3), T cell immunoglobulin domain and mucin domain 3 (TIM3), and sialic acid-binding immunoglobulin-type lectin 7 (SIGLEC7).^270^ Normal physiological context balance costimulatory and coinhibitory signals for maintaining self-tolerance and preventing autoimmunity. However, this balance is perturbed in the TME; coinhibitory signals are used by tumor cells to evade an immune attack. Therefore, costimulatory signal agonists and coinhibitory signal antagonists have been applied in cancer immunotherapy, of which CTLA4, PD-1, and its partner programmed death-ligand 1 (PD-L1) are the most studied.^271^ PD-L1 is a transmembrane ligand expressed on the surface of cancer cells, tumor-infiltrating DCs and macrophages, and binds to PD-1 receptor located in the plasma membrane of cytotoxic CD8^+^ T cells to suppress effector T cell antitumor function.^272^ CTLA4 is a receptor expressed on T_reg_ cells and downregulates immune responses.^273^ Immune checkpoint inhibitors (ICIs) are currently at the forefront of cancer therapy, as they display successful long-lasting antitumor efficacy, even for certain metastatic cancers. However, only 15% of patients respond to immunotherapy.^274^ Anti-PD-1 therapy not only initiates a tumor rejection program, but also induces a competing TGFβ-driven immunosuppressive program.^262^ TGFβ has also been found to drive immune response evasion and attenuate the tumor response to anti-PD-L1 therapy by contributing to the exclusion of CD8^+^ effector T cells.^275,276^ In addition, the unsuccessful antitumor activity of chimeric antigen receptor (CAR) T cell therapy has been attributed in part to TGFβ-induced immunosuppression.^277^ Because of these insights into TGFβ’s immunosuppressive function, the combination of anti-TGFβ therapy and immunotherapy is under intensive investigation.

Many preclinical studies have shown a great success in the combination of immunotherapy with anti-TGFβ therapy (Fig. 6). For example, the TβRI kinase inhibitor galunisertib (LY2157299) has been combined with anti-PD-1/L1 immunotherapy in the treatment of breast cancer in a mouse model. The combined treatment showed an efficient antitumor immunity, leading to more persistent and complete responses.^278^ The anti-CTLA4-TβRII chimera has been studied in preclinical melanoma and TNBC tumor xenograft mouse models and demonstrated superior results with respect to antitumor efficacy than treatment with anti-CTLA4 antibody alone.^279^ The TGFβ ligand trap AVID200 combined with ICIs showed enhanced antitumor immunity in TNBC homograft models.^280^ Recently, researchers engineered a bispecific receptor decoy named CD4 TGFβ Trap that selectively blocks TGF-β signaling in CD4^+^ T cells and promotes reorganization of tumor vasculature and cancer cell death in a mouse mammary tumor virus promoter-driven Polyoma middle T-antigen transgenic mouse model of breast cancer.^281^ SRK-181-mIgG1 is a fully human antibody that selectively binds to latent TGFβ1 and inhibits its activation, which avoided toxicities observed with pan-TGFβ inhibitors.^282^ The coadministration of SRK-181-mIgG1 together with anti-PD-1 inhibitors has been applied in the treatment of checkpoint blockade-resistant syngeneic mouse tumors that displayed an increased influx of intratumoral CD8^+^ T cells and a lower level of immunosuppressive myeloid cells.^282^ Researchers designed pH-responsive clustered nanoparticles that can deliver TβRI kinase inhibitor galunisertib (LY2157299) and small interfering RNA targeting PD-L1 to the PDAC stroma microenvironment, and this regimen showed significant antitumor efficiency by both provoking antitumor immunity and suppressing tumor growth in PDAC mouse models.^283^ However, despite these highly encouraging results mentioned above, we like to provide a note of caution as different tumor models with different immunogenicity can exhibit totally different, sometimes even opposite, results.^284^

Some therapies in which anti-TGFβ and immunotherapy are combined have entered clinical evaluations with patients (Table 1). For example, the TβRI kinase inhibitor vactosertib (TEW-7197) was combined with the anti-PD-L1/PD-1 monoclonal antibody durvalumab in a phase 2 clinical trial for patients with advanced urothelial carcinoma or NSCLC (NCT03732274), and with the anti-PD-1 antibody pembrolizumab in a phase 1/2 clinical evaluation of patients with metastatic colorectal or gastric cancer (NCT03724851). The combination therapies showed a good safety profile and antitumor activity in both trials.^285,286^ SAR439459 is a pan-TGFβ ligand-neutralizing antibody, and the combination of SAR439459 and anti-PD1 antibody lead to tumor regression in the MC38 tumor-bearing mice model.^287^ SAR439459 in combination with the anti-PD1 antibody cemiplimab is now being tested in a phase 1 clinical trials for patients with advanced solid tumors (NCT03192345). NIS793 is a monoclonal antibody blocking TGFβ1 and TGFβ2 and is being tested in combination with the anti-PD1 antibody PDR001 in a phase 1 clinical trial of patients with advanced malignancies (NCT02947165).^262^ M7824 is a bifunctional fusion protein consisting of the ectodomain of TβRII, which serves as a TGFβ ligand trap, and a human monoclonal antibody against PD-L1.^288^ The fusion protein can target both TGFβ- and PD-L1-mediated signaling pathways, and it displayed promising results by eliciting antitumor activity in multiple mouse cancer models.^289^ M7824 showed a manageable safety profile and encouraging clinical efficacy in phase 1 clinical trials, including patients with advanced solid tumors, NSCLCs, recurrent glioblastoma, cervical cancer, metastatic TNBC, heavily pretreated CRC, or human papillomavirus (HPV)-associated cancers.^290–296^ M7824 is now being evaluated in a phase 2 clinical trial for patients with advanced/metastatic biliary tract cancer, gallbladder cancer, recurrent respiratory papillomatosis, thymoma, CRC, head and neck squamous cell cancer, advanced pancreas cancer, or recurrent prostate cancer.^297^ Moreover, the evaluation of M7824 for patients with advanced NSCLC or biliary tract cancer is now under investigation in phase 3 clinical trials.^278,298^ A recent study discovered that an anti-GARP:TGFβ1 mAb (ABBV151), which selectively blocks TGFβ1 production by T_reg_ cells, can induce the regression of anti-PD-1 immunotherapy-resistant tumors in a mouse cancer model.^299,300^ The phase 1 clinical trial of an anti-GARP:TGFβ1 mAb (ABBV151) as monotherapy and in combination with the anti-PD1 mAb budigalimab (ABBV-181) were recently initiated for the evaluation of their safety and tolerability for patients with advanced solid tumors (NCT03821935).

CAR T cell therapy has demonstrated remarkable success by utilizing engineered T cells with tumor antigens that lead to the recognition and attack of tumor cells in blood cancers; however, this method still remains a big challenge for treating solid tumors partially due to TGFβ-mediated immunosuppression.^301^ Inhibiting TGFβ signaling in CART cells can boost their antitumor efficacy in solid tumors.^302,303^ Other strategies also reported overcoming CART cell repression by interfering with TGFβ signaling, such as coexpression of the dominant-negative TβRII,^304^ coexpression of TβRII-41BB to switch the TGFβ-suppressive signal to a 41BB stimulatory signal,^305^ coexpression of TGFβ binding single-chain variable fragment,^306^ and constitutively active AKT.^307^

Anti-TGFβ therapy can also be combined with cancer vaccines to enhance treatment efficacy.^308^ Vaccination against the EMT transcription factor TWIST1 can induce effector T cell responses and suppress murine tumor growth and spontaneous metastasis.^309^ The combination of an adenovirus cancer vaccine encoding tumor-associated antigen TWIST1 (Ad-TWIST) and bifunctional fusion protein M7824 that targets both TGFβ and PD-L1 displayed superior improvement of the antitumor efficacy as compared to Ad-TWIST monotherapy in multiple murine models of human solid tumors.^310^ In addition, a phase 2 clinical trial study of combination therapies with M7824 and therapeutic Tri-Ad (ETBX-011, ETBX-051, and ETBX-061) vaccine for patients with head and neck neoplasms is ongoing (NCT04247282). M7824 has also been combined with the HPV vaccine PRGN-2009 in phase 1/2 clinical trials for subjects with HPV-associated cancers (NCT04432597) and combined with cancer vaccines that target the EMT driver transcription factor brachyury in phase 1/2 clinical trials for patients with advanced breast cancer (NCT04296942) or metastatic castration-resistant prostate cancer (NCT03493945).^291,311,312^

## Concluding remarks

TGFβ was discovered in the late 1970s/early 1980s,^313,314^ and its role as a multifunctional regulator of normal and cancer cell growth became apparent shortly thereafter.^315^ The purification of TGFβ and cloning of its complementary DNA, as well as the identification of TGFβ receptors, paved the way for in vitro and in vivo studies on its mechanism of action and revealed the pleiotropic roles of TGFβ in controlling pathophysiological processes.^1–4,27,55,316–318^ Moreover, these advances allowed the pharmacological interference of key pathways with neutralizing antibodies against TGFβ or TGFβ receptor kinase inhibitors.^319,320^ While preclinical studies in mouse models have shown great promise for TGFβ pharmacological agents, the role of TGFβ as a tumor suppressor and critical role in maintaining tissue homeostasis have made its clinical translation demanding and prevented TGFβ targeting strategies from reaching clinical approval for the treatment of cancer patients. Significant progress has been made to (potentially) make treatment with TGFβ-targeting agents more safer and effective. For example, by performing intermittent dosing strategies bypassing cardiovascular toxicity,^321,322^ by specifically inhibiting only the TGFβ1 isoform that has the strongest link with cancer progression,^282^ or by selecting cancer patients for treatment with a mesenchymal phenotype and high TGFβ activity that are likely to benefit most from the TGFβ targeting.^22,250^

We now know that cancer cells frequently escape from the TGFβ-induced cytostatic response and that TGFβ drives the EMT of cancer cells. Mesenchymal cancer cells have been linked to metastasis and chemotherapy, targeted therapy, and/or radiotherapy resistance. As most cancer patients die of metastasis and demonstrate relapse after chemotherapy, targeted therapy, and/or radiotherapy, the combination of these therapies with TGFβ inhibitors are being tested. Moreover, TGFβ strongly promotes cancer progression by acting on the TME, activating CAFs, stimulating angiogenesis, and eluding the immune system. How TGFβ inhibitors elicit their anticancer effect in patients is frequently unclear, but its reversal of immunosuppressive activity in the TME might be of key importance. Moreover, as the clinical failure of immune checkpoint inhibitors for cancer treatment has been linked to overactive TGFβ signaling activity (at least under some circumstances), we are witnessing a profound renewed interest in TGFβ as a target for cancer therapy. Thus, by combining chemotherapy, targeted therapy, radiotherapy, and immunotherapy with TGFβ-targeting drugs, treatments can be made more efficient by improving antitumor efficacy and reducing therapy resistance. One aspect remains of key importance, finding reliable biomarkers that enable clinicians to select the best (combinatorial) treatment for each individual cancer patient.
